# Modification of Glyceraldehyde-3-Phosphate Dehydrogenase with Nitric Oxide: Role in Signal Transduction and Development of Apoptosis

**DOI:** 10.3390/biom11111656

**Published:** 2021-11-08

**Authors:** Vladimir I. Muronetz, Maria V. Medvedeva, Irina A. Sevostyanova, Elena V. Schmalhausen

**Affiliations:** 1 Belozersky Institute of Physico Chemical Biology, Lomonosov Moscow State University, 119234 Moscow, Russia; irina@belozersky.msu.ru (I.A.S.); shmal@belozersky.msu.ru (E.V.S.); 2 Faculty of Bioengineering and Bioinformatics, Lomonosov Moscow State University, 119234 Moscow, Russia; maria@fbb.msu.ru

**Keywords:** glyceraldehyde-3-phosphate dehydrogenase, *S*-nitrosylation, protein–protein interactions, oxidation, sulfenic acid, NO, apoptosis, Siah1, *S*-glutathionylation

## Abstract

This review focuses on the consequences of GAPDH *S*-nitrosylation at the catalytic cysteine residue. The widespread hypothesis according to which *S*-nitrosylation causes a change in GAPDH structure and its subsequent binding to the Siah1 protein is considered in detail. It is assumed that the GAPDH complex with Siah1 is transported to the nucleus by carrier proteins, interacts with nuclear proteins, and induces apoptosis. However, there are several conflicting and unproven elements in this hypothesis. In particular, there is no direct confirmation of the interaction between the tetrameric GAPDH and Siah1 caused by *S*-nitrosylation of GAPDH. The question remains as to whether the translocation of GAPDH into the nucleus is caused by *S*-nitrosylation or by some other modification of the catalytic cysteine residue. The hypothesis of the induction of apoptosis by oxidation of GAPDH is considered. This oxidation leads to a release of the coenzyme NAD^+^ from the active center of GAPDH, followed by the dissociation of the tetramer into subunits, which move to the nucleus due to passive transport and induce apoptosis. In conclusion, the main tasks are summarized, the solutions to which will make it possible to more definitively establish the role of nitric oxide in the induction of apoptosis.

## 1. Introduction

Hundreds of thousands of articles and tens of thousands of reviews are devoted to the study of the role of nitric oxide as a regulator of the vital activity of all living things, from microorganisms to higher plants and animals. The effect of nitric oxide on proteins has also been studied in sufficient detail, since nitrosylation is one of the most important and widespread post-translational modifications. Sulfhydryl groups of cysteine residues can undergo *S*-nitrosylation to form the corresponding nitrosothiols. *S*-nitrosylation is the covalent attachment of a nitrogen monoxide group to the SH-group of a cysteine residue (Protein-*S*-NO). *S*-nitrosylation is considered to be an important post-translational modification of proteins involved in physiological regulation based on redox potential [1,2,3,4]. Compared to other post-translational modifications, *S*-nitrosothiols are unstable due to the lability of *S*-*N* bonds, the presence of denitrosylases and other reducing agents, such as ascorbate, and the reaction with reduced glutathione (GSH), resulting in the formation of a mixed disulfide between a protein and GSH (*S*-glutathionylation) [5]. The development of methods for the analysis of *S*-nitrosylation of proteins [6,7] has made it possible to identify more than two dozen proteins, the level of *S*-nitrosylation of which changes in some diseases—especially in disorders of the cardiovascular, musculoskeletal, and nervous systems [8,9,10,11,12,13,14,15].

In addition to cysteines, tyrosine residues undergo nitrosylation [16], and one of the consequences of this modification is a crosslinking of proteins due to the formation of dityrosines [17]. However, this type of modification is irreversible, and unlikely to be involved in the regulation of cellular processes, although it can probably contribute to the development of pathological processes. In the present review, we discuss only the nitrosylation of cysteine residues (*S*-nitrosylation).

One of the main targets of NO in cells is glyceraldehyde-3-phosphate dehydrogenase (GAPDH), which is due to the presence of a highly reactive cysteine residue in the active center of this protein, as well as to the high content of this protein in all cells (5–15% of the total amount of soluble proteins of the cytoplasm) [18,19]. Post-translational modifications of GAPDH are of special interest, since they can affect not only the catalytic activity of the enzyme, but also its numerous moonlighting functions, such as oxidative stress response, maintenance of DNA integrity, post-translational gene regulation, protection of telomeric DNA, and the participation of GAPDH in the induction of apoptosis [20]. Modifications of GAPDH can influence its interactions with other proteins and RNA, which affect the development of human disease, tumorigenesis, diabetes, and age-related neurodegenerative disorders [21]. In this review, we focus on the role of *S*-nitrosylation in the regulation of various functions of GAPDH, since other post-translational modifications have been discussed in detail in several recent reviews [3,4,22].

Studies of the effect of nitric oxide on GAPDH have been taking place for almost half a century, but the mechanisms of this modification, and its consequences for the vital activity of the cell, are still far from being fully understood. In 1973, it was first discovered that organic nitrates inhibit the activity of GAPDH and monoamine oxidase—probably due to the modification of sulfhydryl groups important for catalysis [23]. The effect of nitric oxide was studied in more detail in the works of W.S. Allison, who devoted many of his works to studying the role of oxidative modifications of GAPDH, and discovered the appearance of acyl phosphatase activity in this enzyme due to the oxidation of the catalytic cysteine residue to cysteine sulfenic acid (Cys-SOH) [24]. It was shown that the treatment of GAPDH with trinitroglycerin resulted in the disappearance of the main dehydrogenase activity, with simultaneous development of the acyl phosphatase activity [25]. The authors suggested that this modification was due to the oxidation of the catalytic cysteine residue to cysteine sulfenic acid. Treatment of the modified enzyme with ascorbate resulted in the recovery of the dehydrogenase activity and the disappearance of the acyl phosphatase activity, due to the reduction of the cysteine sulfenic acid yielding free cysteine residue. Thus, these studies did not suggest the *S*-nitrosylation of GAPDH, and did not consider a role of such a modification in the regulation of the enzyme function. 

There were two more bursts of interest in the modification of GAPDH with nitric oxide. In 1992, three groups of researchers suggested that NO stimulates ADP-ribosylation of GAPDH [26,27,28]. Then, in 2006, a concept was proposed according to which *S*-nitrosylation of GAPDH is the main link in the NO-induced apoptosis; according to the ideas of this concept’s authors, the incubation of GAPDH with nitric oxide leads to *S*-nitrosylation of the enzyme. The modified enzyme interacts with Siah1 (an E3 ubiquitin ligase), and the formed complex of the two proteins penetrates into the cell nucleus and stimulates a cascade of apoptotic reactions [29]. Both hypotheses, which will be considered in separate sections, stimulated the emergence of new studies related to various aspects of the effect of nitric oxide on GAPDH.

However, there is still no precise information on the effect of *S*-nitrosylation on the enzyme structure, or on the necessity of *S*-nitrosylation of GAPDH for the implementation of the cascade of processes that induce apoptosis. In this review, we will analyze in detail the extensive information on the modification of GAPDH with nitric oxide, and consider the most important issues, without solving which the concept of the role of *S*-nitrosylation of GAPDH in the regulation of cell activity remains incomplete.

## 2. Relationship between ADP-Ribosylation of GAPDH and Its Modification with Nitric Oxide

In 1992, three articles by different research groups reported on the nitric oxide-induced ADP-ribosylation of GAPDH. In the first study, the authors found that upon treatment of erythrocyte membranes with nitroprusside, a radioactive label from [adenylate-^32^P]NAD^+^ was incorporated into a 36 kDa protein. Using monoclonal antibodies, it was proven that the modified protein was GAPDH. The bond between the protein and the nucleotide was destroyed in the presence of HgCl_2_, but was resistant to hydroxylamine. Based on the sensitivity to the effects of these reagents, the authors concluded that ADP-ribosylation occurs at cysteine residues, and not at arginine residues. The stoichiometry of label inclusion was rather low, constituting 8 mmol/mol GAPDH monomer, which excluded a significant effect of this modification on the catalytic activity of the enzyme [26]. In two subsequent papers, it was more definitively concluded that nitric oxide causes ADP-ribosylation of GAPDH and, as a consequence, its inactivation [27,28]. Although the modification affected only ~5% of the total amount of GAPDH present in the studied tissues, the authors suggested an important role of NO-dependent ADP-ribosylation of GAPDH in the regulation of energy metabolism and related processes [28]. Over the course of several years, a series of experiments were carried out to confirm the ADP-ribosylation of GAPDH with the use of radioactively labeled nucleotides, and with biotinylated beta-nicotinamide adenine dinucleotide [30]. The ADP-ribosylated GAPDH was found in various tissues and organisms, and this modification was also shown for other dehydrogenases. It was assumed that GAPDH not only undergoes auto-ADP-ribosylation, but is also capable of catalyzing the ADP-ribosyltransferase reaction. However, even after the first works, there were doubts about the participation of nitric oxide in the ADP-ribosylation of the enzyme. It was shown that ADP-ribosylation of GAPDH is stimulated not only by NO, but also by the enzyme substrate, glyceraldehyde-3-phosphate [31]. The low degree of enzyme modification also did not allow conclusions to be drawn about any important functions of NO-dependent ADP-ribosylation of GAPDH. Several works refuted data on a new type of GAPDH modification. It was proven that the entire NAD^+^ molecule binds to the enzyme, and the nicotinamide fragment is not released into solution. This process is indeed stimulated by nitric oxide, which nitrosylates the cysteine residue in the active center of GAPDH, leading to the inactivation of the enzyme and the covalent binding of NAD^+^. However, the inactivation of GAPDH mainly occurs due to *S*-nitrosylation of the catalytic cysteine residue, since the covalent inclusion of NAD^+^ does not exceed 0.02 mol NAD^+^ per mol GAPDH monomer [32]. The absence of ADP-ribosylation of GAPDH, along with the low efficiency of covalent bonding of NAD^+^ to the enzyme, excluded these modification mechanisms from the list of possible signaling pathways, but led to a more thorough study of the role of *S*-nitrosylation of GAPDH in the regulation of various functions of this enzyme.

## 3. Development of Ideas about the Induction of Apoptosis with the Participation of GAPDH

**Figure 1:**
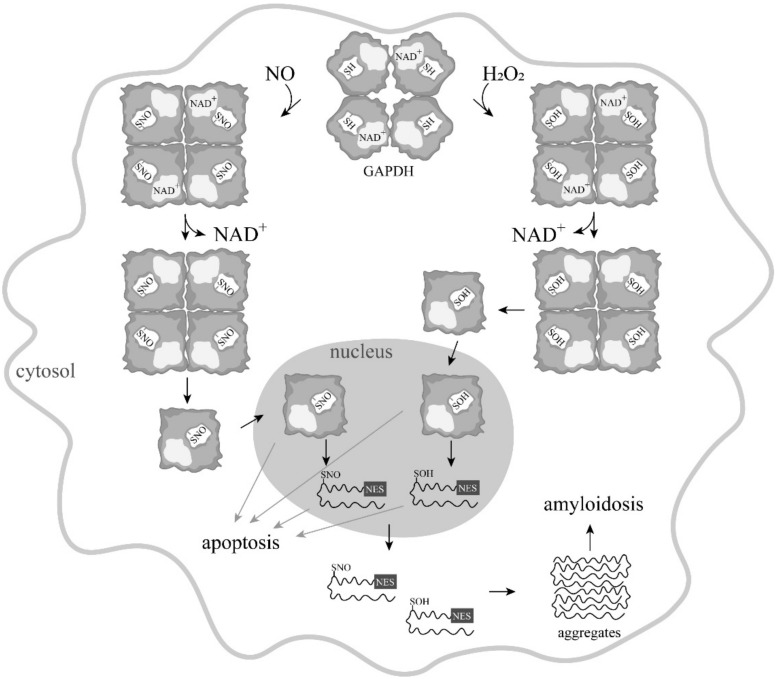
A scheme of apoptosis induced by various modifications of GAPDH. Modifications of the catalytic cysteine in GAPDH decrease the affinity of NAD^+^ for the protein, which results in a release of NAD^+^ from the active center, destabilization of the tetramer, and its dissociation into subunits. The subunits penetrate to the nucleus via passive transport. In the nucleus, unfolding of the GAPDH subunits leads to the exposure of the nuclear export signal (NES) and the subsequent transport of the unfolded GAPDH subunits to the cytoplasm, where they form aggregates (according to [34,35,37,39]).

Since the mid-1990s, information has begun to accumulate about a new function of GAPDH—namely, the participation of this enzyme in the induction of apoptosis. It was shown that in various pathologies, this cytoplasmic enzyme accumulated in the nucleus, which correlated with the induction of apoptosis [33,34]. This accumulation of GAPDH in the nucleus could be caused by exposure to nitric oxide, reactive oxygen species, and other compounds [34,35,36]. It was also assumed that the transfer of GAPDH into the nucleus was due to its ADP-ribosylation, as described in the previous section. Special attention to the nuclear translocation of GAPDH was prompted by the relationship of this phenomenon to the development of neurodegenerative disorders—primarily Parkinson’s disease. Moreover, it was shown that that deprenyl (a drug that ameliorates the progression of Parkinson’s disease) and other structurally related propargylamines bind with GAPDH and increase neuronal survival by interfering with apoptosis signaling pathways [37]. However, ideas about the mechanism of the translocation of GAPDH into the nucleus were contradictory. It is well known that GAPDH is a tetrameric protein of 144 kDa localized in the cytoplasm. At the same time, in many studies on the induction of apoptosis, immunochemical staining detected GAPDH only in the nucleus, but not in the cytoplasm. It was found that this staining was associated with the use of the popular monoclonal antibodies of clone 6C5 against GAPDH, obtained during the first work on NO-dependent ADP-ribosylation [26]. It was later proven that these antibodies do not interact with native tetrameric GAPDH, but recognize partially or fully unfolded forms of the enzyme [38]. Consequently, non-native forms of GAPDH (subunits or unfolded polypeptide chains) were detected in the nucleus. Moreover, the transport of the GAPDH tetramer to the nucleus via carrier proteins is impossible, since the enzyme lacks the nuclear localization signal (NLS). Therefore, the mechanism of GAPDH translocation to the nucleus requires a stage of the dissociation of the oxidized, *S*-nitrosylated, or otherwise modified GAPDH tetramer into subunits. In contrast to the tetrameric molecule, subunits or fully unfolded polypeptide chains of GAPDH can penetrate into the cell nucleus via passive transport [33]. The assumption of the dissociation of a tetrameric molecule into subunits seems logical. It is well known that NAD^+^ is tightly bound in the active center of GAPDH, and exerts a pronounced stabilizing effect on the protein. This tight binding of NAD^+^ is due to the formation of the charge-transfer complex between the nicotinamide ring of the NAD^+^ molecule and the sulfhydryl group of the catalytic cysteine residue in the active center of GAPDH. Oxidation and other modifications of this sulfhydryl group decrease the affinity of NAD^+^ for the protein. Consequently, the release of NAD^+^ from the active center can induce the dissociation of the tetramer into subunits, and their subsequent movement into the nucleus. In addition, the oxidized forms of GAPDH have an increased affinity for nucleic acids, the interaction with which in the nucleus is considered to be one of the mechanisms of apoptosis [39]. The emergence of new data made it possible to refine the scheme of the induction of apoptosis. It was found that GAPDH contains a nuclear export signal (NES) that includes 13 amino acids (KKVVKQASEGPLK) and is located in the C-terminal domain of GAPDH [34]. In native GAPDH, this sequence forms an alpha-helical region on the protein surface and, probably for this reason, is not recognized as an NES by the protein exportin 1, which exports proteins from the nucleus. However, after the translocation of GAPDH from the cytoplasm to the nucleus, the structured regions of the protein unfold, which leads to the exposure of the NES that interacts with the exporter proteins. The deletion of the NES sequence, or the introduction of a mutation in it, increased the accumulation of GAPDH in the nucleus. Based on these results, it was concluded that unfolded polypeptide chains cannot accumulate in the nucleus, as suggested previously. A new version of the scheme suggested that as a result of various effects, GAPDH dissociates into compact subunits in which the NES is structured, and after their transport to the nucleus, the protein unfolds and the NES is exposed (Figure 1). The fully unfolded polypeptide chains, due to the presence of the NES in them, again move into the cytoplasm [34].

**Figure 2:**
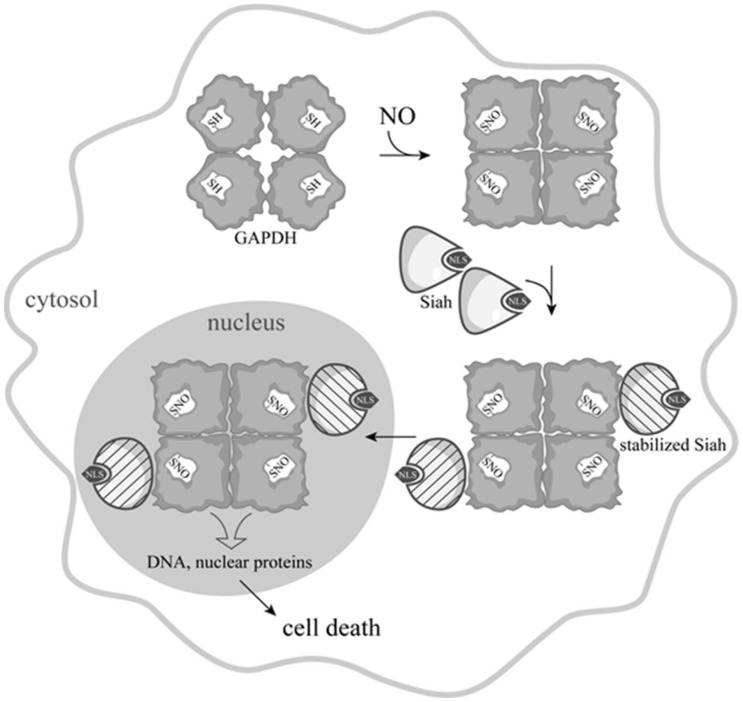
Scheme of NO-induced apoptosis mediated by GAPDH proposed by A. Sawa [49], with the depiction of the tetrameric GAPDH structure. *S*-nitrosylation of GAPDH promotes its binding with Siah1. Siah1, which possesses a nuclear localization signal (NLS), translocates GAPDH to the nucleus. In the nucleus, GAPDH interacts with nuclear proteins, leading to apoptosis.

All schemes and hypotheses regarding the translocation of GAPDH into the nucleus were discarded after the emergence of the works by the group of S. Snyder and A. Sawa on the role of *S*-nitrosylation of GAPDH in the induction of apoptosis. As noted in the previous section, the functional role of the ADP-ribosylation by nitric oxide was not confirmed, but more and more evidence testified to the importance of the *S*-nitrosylation of GAPDH by nitric oxide. Most of the works were related to the study of the NO-induced apoptosis mediated by GAPDH, as well as to the relationship between *S*-nitrosylation of GAPDH and the development of neurodegenerative diseases—primarily Parkinson’s disease [40,41,42,43,44]. In addition, the changes in the catalytic activity of *S*-nitrosylated GAPDH, along with the possible effect of this modification on the energy metabolism of cells, were investigated [45,46,47]. It was also found that *S*-nitrosylation decreases the affinity of GAPDH for the band 3 protein of the erythrocyte membranes, which interacts not only with GAPDH, but also with other proteins of glycolysis [48]. All of this indicated the important role of *S*-nitrosylation of GAPDH in the regulation of energy metabolism and the vital activity of the cell as a whole. However, it became more and more difficult to reconcile all of the facts with one another. The main contradiction was that in order to perform various unusual functions in the nucleus, GAPDH must retain its structure. This would allow the enzyme to modify other proteins and/or form complexes with them, regulating their functions. In addition, only maintaining the spatial structure of GAPDH would prevent its export from the nucleus, due to NES shielding. However, the tetrameric molecule is unable to penetrate the nucleus, due to its large size and lack of an NLS. A series of elegant works by the group of S. Snyder and A. Sawa resolved these contradictions, suggesting a completely new signaling mechanism, the key element of which was *S*-nitrosylation of GAPDH (Figure 2).

According to this mechanism, a wide range of apoptotic stimuli augment NO production via the induction of inducible nitric oxide synthase (iNOS), or by the activation of neuronal nitric oxide synthase (nNOS). GAPDH is nitrosylated by NO at the catalytic cysteine residue, which leads to inactivation of the protein and to conformational changes in its molecule. These alterations facilitate the binding of GAPDH with the E3-ubiquitin-ligase Siah1. Siah1, which possesses a nuclear localization signal (NLS), translocates GAPDH to the nucleus. It was found that lysine 225 in the GAPDH sequence is critical for its binding with Siah1, since the K225 mutation prevents nuclear translocation of both GAPDH and Siah1. The interaction with GAPDH results in the stabilization of Siah1, which enables rapid degradation of nuclear proteins, leading to cell death [49,50,51,52]. According to a later version of this mechanism, nuclear GAPDH is acetylated at Lys 160 by the acetyltransferase p300/CBP (CREB-binding protein) via direct protein interaction which, in turn, stimulates the acetylation and catalytic activity of p300/CBP. The downstream targets of p300/CBP—such as p53—are activated, and cause cell death [53].

In a subsequent work by the same group, this signaling pathway was complicated due to the identification of one more protein. It was shown that, in addition to Siah1, GAPDH interacts with a cytoplasmic protein of 52 kDa. The binding of this protein to GAPDH interferes with its interaction with Siah1, and prevents apoptosis. This protein was named GOSPEL (GAPDH’s competitor of Siah Protein Enhances Life) for its function in this signaling pathway [54]. It was found that *S*-nitrosylation of GOSPEL significantly enhances its binding to GAPDH and, as a consequence, prevents the translocation of GAPDH into the nucleus. It should be noted that GOSPEL could bind to both *S*-nitrosylated and unmodified GAPDH. Moreover, the exposure to NO results in *S*-nitrosylation of both GAPDH and GOSPEL, which simultaneously stimulates the interaction of GAPDH-SNO with Siah1 and GOSPEL-SNO with GAPDH, so that the two processes (inducing and preventing apoptosis) compete with one another. The authors explained this contradiction based on the fact that *S*-nitrosylation of these two proteins is separated in time: first, GOSPEL is modified, and then GAPDH. Thus, with a sufficient content of GOSPEL in cells, the induction of apoptosis does not occur. This hypothesis was not developed further. Moreover, it was shown that GOSPEL stimulates the formation of amyloid aggregates induced by the *S*-nitrosylation of GAPDH [55]. Then, one more mechanism of signal transduction mediated by *S*-nitrosylated GAPDH was proposed, according to which the nitric oxide group is transferred from *S*-nitrosylated GAPDH to nuclear proteins, including the deacetylating enzyme sirtuin-1 (SIRT1), histone deacetylase-2 (HDAC2), and DNA-activated protein kinase (DNA-PK) [56]. It is likely that the same pathway involving the *S*-nitrosylation of GAPDH and SirT1 leads to the stimulation of the acetylation of the tau protein involved in the development of Alzheimer’s disease [13]. According to the results obtained in another research group using a rat stroke model, nuclear GAPDH that translocates to the nucleus under oxidative/nitrosative stress mediates brain damage by binding with nuclear poly(ADP-ribose) polymerase-1 (PARP-1). According to the suggested mechanism, the N-terminus of nuclear GAPDH binds with PARP-1, and this complex promotes PARP-1 overactivation, leading to brain damage and neurological deficits both in vitro and in vivo [57].

In parallel with the study of the role of nuclear GAPDH in the development of apoptosis, the role of the formation of GAPDH aggregates in the cytoplasm was investigated. It was suggested that unfolded GAPDH subunits could participate in the formation of amyloid structures, which are characteristic of neurodegenerative disorders [58,59]. It was shown that amyloid-like structures containing GAPDH polypeptide chains crosslinked by disulfide bonds can form under oxidative stress [60,61]. It was assumed that the amyloid-like GAPDH aggregates could induce apoptosis and other disorders of cellular functions. This assumption was supported by the fact that compounds preventing GAPDH aggregation exhibited anti-apoptotic effects [62,63]. It was shown that extracellular complexes of GAPDH and beta-amyloid peptide exhibit high cytotoxicity, and may be one of the causes of neurodegenerative changes in Alzheimer’s disease [64]. The data on the colocalization of GAPDH and alpha-synuclein in Lewy bodies (structures that are characteristic of Parkinson’s disease) [65], together with the data on the interaction between GAPDH and alpha-synuclein in vitro [66], point to a possible involvement of GAPDH in the development of Parkinson’s disease.

## 4. Induction of Apoptosis by Oxidation and Other Modifications of the Catalytic Cysteines of GAPDH

Stimulation of apoptosis by nitric oxide, mediated by the movement of GAPDH from the cytoplasm to the nucleus, is beyond doubt. In many studies, it has been shown that exposure to nitric oxide leads to the accumulation of GAPDH in the cell nucleus, where it interacts with both DNA and various proteins, initiating a cascade of reactions that induce apoptosis [50,52,53]. However, there is still no rigorous evidence that the transport of GAPDH into the nucleus and its interaction with other proteins occurs only if GAPDH undergoes *S*-nitrosylation at the catalytic cysteine residues. Several facts indicate that *S*-nitrosylation of GAPDH is, at least, not the only mechanism for the induction of apoptosis with the participation of GAPDH [36].

**Figure 3:**
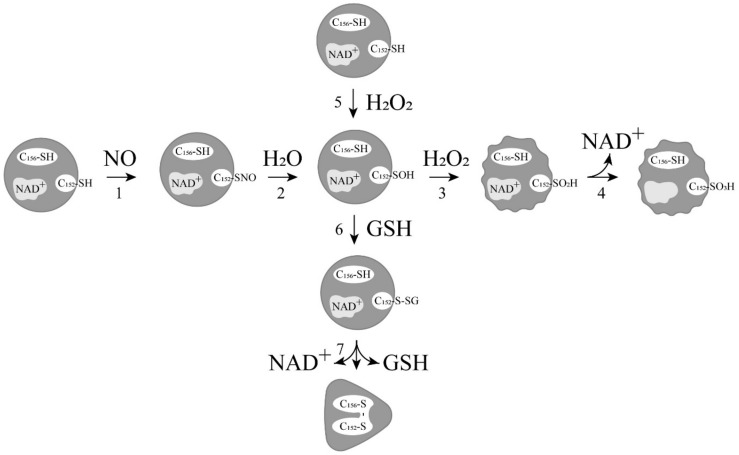
Relationships between different modifications of GAPDH. One of four identical subunits is shown. C_152_ is the catalytic cysteine residue; C_156_ is the cysteine residue that is not involved in catalysis.

As already noted, there are a number of works that report the appearance of cysteine sulfenic acid (Cys-SOH) in GAPDH after *S*-nitrosylation. One of the first works reported the formation of cysteine sulfenic acid in the active center of GAPDH after incubation with trinitroglycerin. The modification of the catalytic cysteine of GAPDH yielding the cysteine sulfenic acid resulted in the loss of the dehydrogenase activity, but endowed the enzyme with the ability to break down acylphosphates (acylphosphatase activity) [25]. Later, a similar effect was observed after the treatment of GAPDH with the NO donors *S*-nitrosoglutathione, 3-morpholinosydnonimine, and diethylamine NONOate [67]. More, the mass spectrometric analysis of nuclear GAPDH following apoptotic stimulation revealed sulfonation (Cys-SO_3_H) at the catalytic cysteine residue. It was assumed that the sulfonation can arise following *S*-nitrosylation of GAPDH cysteines, with subsequent hydrolysis of Cys-SNO to Cys-SOH, and its subsequent oxidation to cysteine sulfinic (Cys-SO_2_H) and then cysteine sulfonic acid [49]. In our recent study, MALDI and ESI mass spectrometry analysis showed that *S*-nitrosylation of GAPDH at the catalytic cysteine residue results in the formation of GAPDH-SNO and GAPDH-SOH [68]. Consequently, it has been confirmed that *S*-nitrosylation of GAPDH results in the oxidation of the catalytic cysteine, yielding the relatively stable cysteine sulfenic acid, which cannot exclude its further oxidation (pathways 1–4 in Figure 3).

Consequently, we cannot exclude the possibility that GAPDH with oxidized sulfhydryl groups moves into the nucleus and causes a cascade of reactions, leading to apoptosis. This assumption is confirmed by a number of observations on the direct induction of apoptosis by hydrogen peroxide. Thus, it was shown that the addition of hydrogen peroxide to Hela cells stimulates the accumulation of non-native forms of GAPDH in the nucleus, and induces cell death [36]. The absence of an NLS in GAPDH, and the large size of the protein, excludes its translocation into the nucleus in the native state. It was assumed that the oxidation of cysteine residues leads to a weakening of NAD^+^ binding in the active center of the enzyme, which results in its dissociation from the active center, decreasing the stability of the tetrameric molecule. Monomeric subunits of GAPDH of 36 kDa can penetrate into the nucleus due to passive transport (Figure 1). In the nucleus, further unfolding of subunits and exposure of the nuclear export signal (NES) take place. Such unfolded GAPDH subunits are transported into the cytoplasm or released from the cells broken during apoptosis, aggregate, and exert a toxic effect on cells unaffected by apoptosis.

It should also be noted that oxidation of GAPDH is closely related to *S*-glutathionylation. It was shown that the oxidation of GAPDH by hydrogen peroxide in the presence of GSH in vitro stimulates *S*-glutathionylation of the catalytic cysteine of GAPDH. The *S*-glutathionylated GAPDH (GAPDH-SSG) is formed due to the reaction between GSH and a sulfenic acid derivative of the catalytic cysteine residue (Cys150 in rabbit or Cys152 in human GAPDH) (Figure 3, reactions 5 and 6). The mixed disulfide (GAPDH-SSG) can then react with the nearby non-catalytic cysteine (Cys154 in rabbit or Cys156 in human), yielding an intrasubunit disulfide bond in the active center of GAPDH (Figure 3, reaction 7). This leads to the dissociation of NAD^+^ from the active center of GAPDH, and significantly decreases the thermal stability of the protein, indicating conformational changes in the enzyme molecule. These changes may affect interactions between GAPDH and proteins and ligands [4,69,70]. Considering that the concentration of GSH in cells is maintained at a high level, the oxidation of the catalytic cysteines to cysteine sulfenic acid should lead to the *S*-glutathionylation of GAPDH. This assumption is supported by the data on the increase in *S*-glutathionylated GAPDH in human endothelial cells after treatment with H_2_O_2_ [71]. Moreover, recently, it has been shown that exposure of HEK 293T cells to hydrogen peroxide leads to rapid *S*-glutathionylation and nuclear translocation of GAPDH. The intrasubunit C152-C156 disulfide bond in the active site was also detected in the nuclear GAPDH. The nuclear GAPDH forms a protein complex with deacetylase SirT1, resulting in *S*-glutathionylation of SirT1 and inhibition of the deacetylase activity. Inactivated SirT1 forms a stable complex with acetylated-p53, and initiates apoptotic signaling, resulting in cleavage of caspase-3. The authors suggest the GAPDH/SirT1/p53 pathway as a common apoptotic mechanism [72].

Consequently, although nitric oxide leads to *S*-nitrosylation of GAPDH, other modified forms (e.g., oxidized and *S*-glutathionylated) can also participate in the subsequent signaling pathway, since there are no direct data on the selective interaction of the *S*-nitrosylated GAPDH (GAPDH-SNO) with partner proteins. Most likely, any modifications of the catalytic cysteine residue, which are accompanied by a weakening of interactions between the enzyme and the cofactor NAD^+^, lead to a change in the conformation of GAPDH, and stimulate its binding to some partner proteins—in particular, Siah1.

## 5. Is GAPDH-SNO an Obligatory Participant in NO-Induced Apoptosis?

A new pathway for the induction of apoptosis, discovered in the works of S. Snyder and A. Sawa, first demonstrated the relationship of GAPDH modification by nitric oxide with the subsequent cascade of reactions leading to programmed cell death [29,49,51,52,53]. Numerous elegant experiments carried out by these researchers have led to the widespread hypothesis that the interaction of GAPDH-SNO with Siah1 (an E3 ubiquitin ligase) is a key element in signal transduction from NO-producing systems to proteins that induce apoptosis. However, some elements of this path are still not fully explored.

First of all, it should be noted that there is still no direct evidence on the interaction of the native tetrameric GAPDH molecule with Siah1, nor on the enhancement of this interaction after *S*-nitrosylation of GAPDH. The proposed concept is based on the formation of a complex between the GAPDH tetramer of 144 kDa and Siah1 containing a nuclear localization signal (NLS) [49]. However, it remains unclear whether it is a tetrameric GAPDH molecule that is involved in this signaling pathway, or whether Siah1 interacts with a separate polypeptide chain of GAPDH. It should be noted that the interaction between GAPDH and Siah1 revealed in the two-hybrid yeast system [52] is typical even for unmodified GAPDH. In this study, lysine 225 in GAPDH was critical for the binding between GAPDH and Siah1. The GAPDH used in the work contained an additional 10-amino-acid sequence of the Myc tag of 1202 Da, the presence of which could prevent the formation of the native tetrameric structure. Thus, all of the effects observed during *S*-nitrosylation could be explained by the interaction between Siah1 and partially unfolded GAPDH subunits. Of course, in this case, the presence of the NLS in Siah1 would give advantages in the transport of GAPDH into the cell nucleus, as compared to free subunits. Since each of the four GAPDH subunits possesses an active center and, under certain conditions, monomers of the enzyme have dehydrogenase activity, they could also retain certain functions in the nucleus (to *S*-nitrosylate or *S*-glutathionylate partner proteins, resulting in the induction of apoptosis).

There is one more question that arises about the induction of apoptosis by nitric oxide according to the described hypothesis: the mechanism of the influence of *S*-nitrosylation of GAPDH on its ability to interact with Siah1 is unclear. Unfortunately, despite the popularity of the proposed hypothesis, thus far there have been no direct experiments to study the change in the affinity between these two proteins upon *S*-nitrosylation of GAPDH. Numerous experiments on cell lines [52,54] only indirectly indicate this possibility. As mentioned above, the selective binding between the *S*-nitrosylated tetramer GAPDH and Siah1 seems unlikely. It is known that the conformation of GAPDH changes upon its interaction with the cofactors NAD and NADH, as well as upon modification of the catalytic cysteine residue. Thus, a change in the conformation of GAPDH upon interaction with various ligands affects its ability to interact with other proteins (for example, with glycolysis partners, as well as structural and membrane proteins). However, the assumption that it is *S*-nitrosylation that stimulates the binding of GAPDH to Siah1, while other modifications of the catalytic cysteine residue—primarily oxidation—do not exert such stimulation, requires direct confirmation.

Due to the lack of unambiguous evidence for the involvement of *S*-nitrosylated GAPDH at all stages of the NO-induced signaling pathway leading to apoptosis, the prevalence of this pathway is questioned. It is possible that *S*-nitrosylation under the action of nitric oxide is an initial apoptotic signal, and that other modified forms of GAPDH—primarily oxidized forms—are involved in the cascade of reactions that induce apoptosis.

## 6. Conclusions

The induction of apoptosis by NO through a signaling pathway, the key element of which is glyceraldehyde-3-phosphate dehydrogenase, has been confirmed in many studies and is beyond doubt. However, the molecular mechanisms of this signaling pathway have not yet been studied in detail, and the proposed hypotheses are based on conflicting experimental data. To substantiate the mechanisms of the participation of GAPDH in the induction of apoptosis under the action of nitric oxide, it is necessary to solve the following problems:

To investigate changes in GAPDH structure caused by *S*-nitrosylation, and to study how these changes affect the strength of its binding to partner proteins—namely Siah1, GOSPEL, Sirt1 and others.

- To isolate, or obtain from isolated proteins, complexes of *S*-nitrosylated GAPDH with partner proteins, and prove that the tetrameric GAPDH molecule is capable of forming such complexes;
- To reveal the presence of tetrameric GAPDH molecules in the nucleus;
- To study the possibility of the formation of complexes between *S*-nitrosylated, oxidized, and *S*-glutathionylated GAPDH with the partner proteins;
- To prove or disprove the possibility of participation of the oxidized or *S*-glutathionylated GAPDH in the induction of apoptosis.

Solving these problems will make it possible to elucidate the molecular mechanisms of the NO-induced apoptosis with the participation of GAPDH, and to find new targets for the action of anti-apoptotic compounds. These studies are of particular importance for the prevention and treatment of neurodegenerative diseases—primarily Parkinson’s disease, for which GAPDH is shown to be directly involved in apoptotic changes in the cells of the substantia nigra.

## References

[1] Hess D.T., Matsumoto A., Kim S.O., Marshall H.E., Stamler J.S. (2005). Protein S-nitrosylation: Purview and parameters. Nat. Rev. Mol. Cell Biol..

[2] Foster M.W., Hess D.T., Stamler J.S. (2009). Protein S-nitrosylation in health and disease: A current perspective. Trends Mol. Med..

[3] Tossounian M.A., Zhang B., Gout I. (2020). The writers, readers, and erasers in redox regulation of GAPDH. Antioxidants.

[4] Muronetz V.I., Melnikova A.K., Saso L., Schmalhausen E.V. (2020). Influence of Oxidative Stress on Catalytic and Non-glycolytic Functions of Glyceraldehyde-3-phosphate Dehydrogenase. Curr. Med. Chem..

[5] Martínez-Ruiz A., Araújo I.M., Izquierdo-Álvarez A., Hernansanz-Agustín P., Lamas S., Serrador J.M. (2013). Specificity in S-nitrosylation: A short-range mechanism for NO signaling?. Antioxid. Redox Signal..

[6] Jaffrey S.R., Erdjument-Bromage H., Ferris C.D., Tempst P., Snyder S.H. (2001). Protein S-nitrosylation: A physiological signal for neuronal nitric oxide. Nat. Cell Biol..

[7] Gow A.J., Chen Q., Hess D.T., Day B.J., Ischiropoulos H., Stamler J.S. (2002). Basal and Stimulated Protein S-Nitrosylation in Multiple Cell Types and Tissues. J. Biol. Chem..

[8] Gekeler V., Weger S., Probst H. (1990). Mdr1P-glycoprotein gene segments analyzed from various human leukemic cell lines exhibiting different multidrug resistance profiles. Biochem. Biophys. Res. Commun..

[9] Zhang Y., Deng Y., Yang X., Xue H., Lang Y. (2020). The Relationship Between Protein S-Nitrosylation and Human Diseases: A Review. Neurochem. Res..

[10] Meneghetti E., Gasperini L., Virgilio T., Moda F., Tagliavini F., Benetti F., Legname G. (2019). Prions Strongly Reduce NMDA Receptor S-Nitrosylation Levels at Pre-symptomatic and Terminal Stages of Prion Diseases. Mol. Neurobiol..

[11] Zahid S., Khan R., Oellerich M., Ahmed N., Asif A.R. (2014). Differential S-nitrosylation of proteins in Alzheimer’s disease. Neuroscience.

[12] Nakamura T., Oh C., Zhang X., Tannenbaum S.R., Lipton S.A. (2021). Protein Transnitrosylation Signaling Networks Contribute to Inflammaging and Neurodegenerative Disorders. Antioxid. Redox Signal..

[13] Sen T., Saha P., Sen N. (2018). Nitrosylation of GAPDH augments pathological tau acetylation upon exposure to amyloid-β. Sci. Signal..

[14] Rizza S., Cardaci S., Montagna C., Di Giacomo G., De Zio D., Bordi M., Maiani E., Campello S., Borreca A., Puca A.A. (2018). S-nitrosylation drives cell senescence and aging in mammals by controlling mitochondrial dynamics and mitophagy. Proc. Natl. Acad. Sci. USA.

[15] Chung K.K.K., Dawson T.M., Dawson V.L. (2005). Nitric oxide, S-nitrosylation and neurodegeneration. Cell. Mol. Biol..

[16] Zhan X., Huang Y., Qian S. (2018). Protein Tyrosine Nitration in Lung Cancer: Current Research Status and Future Perspectives. Curr. Med. Chem..

[17] Van Maarschalkerweerd A., Pedersen M.N., Peterson H., Nilsson M., Nguyen T.T.T., Skamris T., Rand K., Vetri V., Langkilde A.E., Vestergaard B. (2015). Formation of covalent di-tyrosine dimers in recombinant α-synuclein. Intrinsically Disord. Proteins.

[18] Seidler N.W. (2013). GAPDH: Biological Properties and Diversity.

[19] Harris J.I., Waters M., Boyer P.D. (1976). Glyceraldehyde 3-Phosphate Dehydrogenase. The Enzymes.

[20] Sirover M.A. (2011). On the functional diversity of glyceraldehyde-3-phosphate dehydrogenase: Biochemical mechanisms and regulatory control. Biochim. Biophys. Acta..

[21] Sirover M.A. (2020). Moonlighting glyceraldehyde-3-phosphate dehydrogenase: Posttranslational modification, protein and nucleic acid interactions in normal cells and in human pathology. Crit. Rev. Biochem. Mol. Biol..

[22] Sirover M.A. (2021). The role of posttranslational modification in moonlighting glyceraldehyde-3-phosphate dehydrogenase structure and function. Amino Acids.

[23] Jakschik S.B., Needleman P. (1973). Sulfhydryl reactivity of organic nitrates: Biochemical basis for inhibition of glyceraldehyde-P dehydrogenase and monoamine oxidase. Biochem. Biophys. Res. Commun..

[24] Allison W.S., Connors M.J. (1970). The activation and inactivation of the acyl phosphatase activity of glyceraldehyde-3-phosphate dehydrogenase. Arch. Biochem. Biophys..

[25] You K.-S., Benitez L.V., McConachie W.A., Allison W.S. (1975). The conversion of glyceraldehyde-3-phosphate dehyrogenase to an acylphosphatase by trinitroglycerin and inactivation of this activity by azide and ascorbate. Biochim. Biophys. Acta-Enzymol..

[26] Kots A.Y., Skurat A.V., Sergienko E.A., Bulargina T.V., Severin E.S. (1992). Nitroprusside stimulates the cysteine-specific mono(ADP-ribosylation) of glyceraldehyde-3-phosphate dehydrogenase from human erythrocytes. FEBS Lett..

[27] Dimmeler S., Lottspeich F., Brüne B. (1992). Nitric oxide causes ADP-ribosylation and inhibition of glyceraldehyde-3-phosphate dehydrogenase. J. Biol. Chem..

[28] Zhang J., Snyder S.H. (1992). Nitric oxide stimulates auto-ADP-ribosylation of glyceraldehyde-3-phosphate dehydrogenase. Proc. Natl. Acad. Sci. USA.

[29] Hara M.R., Snyder S.H. (2006). Nitric Oxide–GAPDH–Siah: A Novel Cell Death Cascade. Cell. Mol. Neurobiol..

[30] Zhang J., Snyder S.H. (1993). Purification of a nitric oxide-stimulated ADP-ribosylated protein using biotinylated. beta.-nicotinamide adenine dinucleotide. Biochemistry.

[31] Kots A.Y., Sergienko E.A., Bulargina T.V., Severin E.S. (1993). Glyceraldehyde-3-phosphate activates auto-ADP-ribosylation of glyceraldehyde-3-phosphate dehydrogenase. FEBS Lett..

[32] McDonald L.J., Moss J. (1993). Stimulation by nitric oxide of an NAD linkage to glyceraldehyde-3-phosphate dehydrogenase. Proc. Natl. Acad. Sci. USA.

[33] Carlile G.W., Chalmers-Redman R.M., Tatton N.A., Borden K.E., Tatton W.G. (2001). Reduced apoptosis after nerve growth factor and serum withdrawal: Conversion of tetrameric glyceraldehyde-3-phosphate dehydrogenase to a dimer. Mol. Pharmacol..

[34] Brown V.M., Krynetski E.Y., Krynetskaia N.F., Grieger D., Mukatira S.T., Murti K.G., Slaughter C.A., Park H.-W., Evans W.E. (2004). A Novel CRM1-mediated Nuclear Export Signal Governs Nuclear Accumulation of Glyceraldehyde-3-phosphate Dehydrogenase following Genotoxic Stress. J. Biol. Chem..

[35] Dastoor Z., Dreyer J.L. (2001). Potential role of nuclear translocation of glyceraldehyde-3-phosphate dehydrogenase in apoptosis and oxidative stress. J. Cell Sci..

[36] Arutyunova E.I., Domnina L.V., Chudinova A.A., Makshakova O.N., Arutyunov D.Y., Muronetz V.I. (2013). Localization of non-native D-glyceraldehyde-3-phosphate dehydrogenase in growing and apoptotic HeLa cells. Biochemistry.

[37] Tatton W., Chalmers-Redman R., Tatton N. (2003). Neuroprotection by deprenyl and other propargylamines: Glyceraldehyde-3-phosphate dehydrogenase rather than monoamine oxidase B. J. Neural Transm..

[38] Grigorieva J.A., Dainiak M.B., Katrukha A.G., Muronetz V.I. (1999). Antibodies to the Nonnative Forms of d-Glyceraldehyde-3-Phosphate Dehydrogenase: Identification, Purification, and Influence on the Renaturation of the Enzyme. Arch. Biochem. Biophys..

[39] Arutyunova E.I., Danshina P.V., Domnina L.V., Pleten A.P., Muronetz V.I. (2003). Oxidation of glyceraldehyde-3-phosphate dehydrogenase enhances its binding to nucleic acids. Biochem. Biophys. Res. Commun..

[40] Benhar M., Stamler J.S. (2005). A central role for S-nitrosylation in apoptosis. Nat. Cell Biol..

[41] Sultana R., Poon H.F., Cai J., Pierce W.M., Merchant M., Klein J.B., Markesbery W.R., Butterfield D.A. (2006). Identification of nitrated proteins in Alzheimer’s disease brain using a redox proteomics approach. Neurobiol. Dis..

[42] Duncan A., Heales S. (2005). Nitric oxide and neurological disorders. Mol. Aspects Med..

[43] Borutaite V., Brown G.C. (2003). Nitric oxide induces apoptosis via hydrogen peroxide, but necrosis via energy and thiol depletion. Free Radic. Biol. Med..

[44] Brüne B., Lapetina E.G. (1995). Protein thiol modification of glyceraldehyde-3-phosphate dehydrogenase as a target for nitric oxide signaling. Genet. Eng..

[45] Brune B., Mohr S. (2001). Protein Thiol Modification of Glyceraldehyde-3-phosphate Dehydrogenase and Caspase-3 by Nitric Oxide. Curr. Protein Pept. Sci..

[46] Galli F., Rossi R., Di Simplicio P., Floridi A., Canestrari F. (2002). Protein Thiols and Glutathione Influence the Nitric Oxide-Dependent Regulation of the Red Blood Cell Metabolism. Nitric Oxide.

[47] Borderie D. (2000). Nitric Oxide Modifies Glycolytic Pathways in Cultures Human Synoviocytes. Cell Biol. Int..

[48] Galli F., Rovidati S., Ghibelli L., Canestrari F. (1998). S-Nitrosylation of Glyceraldehyde-3-Phosphate Dehydrogenase Decreases the Enzyme Affinity to the Erythrocyte Membrane. Nitric Oxide.

[49] Hara M.R., Cascio M.B., Sawa A. (2006). GAPDH as a sensor of NO stress. Biochim. Biophys. Acta-Mol. Basis Dis..

[50] Hara M.R., Snyder S.H. (2007). Cell signaling and neuronal death. Annu. Rev. Pharmacol. Toxicol..

[51] Hara M.R., Thomas B., Cascio M.B., Bae B.-I., Hester L.D., Dawson V.L., Dawson T.M., Sawa A., Snyder S.H. (2006). Neuroprotection by pharmacologic blockade of the GAPDH death cascade. Proc. Natl. Acad. Sci. USA.

[52] Hara M.R., Agrawal N., Kim S.F., Cascio M.B., Fujimuro M., Ozeki Y., Takahashi M., Cheah J.H., Tankou S.K., Hester L.D. (2005). S-nitrosylated GAPDH initiates apoptotic cell death by nuclear translocation following Siah1 binding. Nat. Cell Biol..

[53] Sen N., Hara M.R., Kornberg M.D., Cascio M.B., Bae B.-I., Shahani N., Thomas B., Dawson T.M., Dawson V.L., Snyder S.H. (2008). Nitric oxide-induced nuclear GAPDH activates p300/CBP and mediates apoptosis. Nat. Cell Biol..

[54] Sen N., Hara M.R., Ahmad A.S., Cascio M.B., Kamiya A., Ehmsen J.T., Aggrawal N., Hester L., Doré S., Snyder S.H. (2009). GOSPEL: A Neuroprotective Protein that Binds to GAPDH upon S-Nitrosylation. Neuron.

[55] González M.C., Romero J.M., Ingaramo M.C., Muñoz Sosa C.J., Curtino J.A., Carrizo M.E. (2016). Enhancement by GOSPEL protein of GAPDH aggregation induced by nitric oxide donor and its inhibition by NAD ^+^. FEBS Lett..

[56] Kornberg M.D., Sen N., Hara M.R., Juluri K.R., Nguyen J.V.K., Snowman A.M., Law L., Hester L.D., Snyder S.H. (2010). GAPDH mediates nitrosylation of nuclear proteins. Nat. Cell Biol..

[57] Nakajima H., Kubo T., Ihara H., Hikida T., Danjo T., Nakatsuji M., Shahani N., Itakura M., Ono Y., Azuma Y.-T. (2015). Nuclear-translocated Glyceraldehyde-3-phosphate Dehydrogenase Promotes Poly(ADP-ribose) Polymerase-1 Activation during Oxidative/Nitrosative Stress in Stroke. J. Biol. Chem..

[58] Naletova I., Schmalhausen E., Kharitonov A., Katrukha A., Saso L., Caprioli A., Muronetz V. (2008). Non-native glyceraldehyde-3-phosphate dehydrogenase can be an intrinsic component of amyloid structures. Biochim. Biophys. Acta..

[59] Muronetz V.I., Barinova K.V., Stroylova Y.Y., Semenyuk P.I., Schmalhausen E.V. (2017). Glyceraldehyde-3-phosphate dehydrogenase: Aggregation mechanisms and impact on amyloid neurodegenerative diseases. Int. J. Biol. Macromol..

[60] Nakajima H., Amano W., Fujita A., Fukuhara A., Azuma Y.-T., Hata F., Inui T., Takeuchi T. (2007). The Active Site Cysteine of the Proapoptotic Protein Glyceraldehyde-3-phosphate Dehydrogenase Is Essential in Oxidative Stress-induced Aggregation and Cell Death. J. Biol. Chem..

[61] Nakajima H., Amano W., Kubo T., Fukuhara A., Ihara H., Azuma Y.-T., Tajima H., Inui T., Sawa A., Takeuchi T. (2009). Glyceraldehyde-3-phosphate dehydrogenase aggregate formation participates in oxidative stress-induced cell death. J. Biol. Chem..

[62] Itakura M., Nakajima H., Semi Y., Higashida S., Azuma Y.-T., Takeuchi T. (2015). Glyceraldehyde-3-phosphate dehydrogenase aggregation inhibitor peptide: A potential therapeutic strategy against oxidative stress-induced cell death. Biochem. Biophys. Res. Commun..

[63] Lazarev V.F., Nikotina A.D., Semenyuk P.I., Evstafyeva D.B., Mikhaylova E.R., Muronetz V.I., Shevtsov M.A., Tolkacheva A.V., Dobrodumov A.V., Shavarda A.L. (2016). Small molecules preventing GAPDH aggregation are therapeutically applicable in cell and rat models of oxidative stress. Free Radic. Biol. Med..

[64] Lazarev V.F., Tsolaki M., Mikhaylova E.R., Benken K.A., Shevtsov M.A., Nikotina A.D., Lechpammer M., Mitkevich V.A., Makarov A.A., Moskalev A.A. (2021). Extracellular GAPDH Promotes Alzheimer Disease Progression by Enhancing Amyloid-β Aggregation and Cytotoxicity. Aging Dis..

[65] Mazzola J.L., Sirover M.A. (2002). Alteration of intracellular structure and function of glyceraldehyde-3-phosphate dehydrogenase: A common phenotype of neurodegenerative disorders?. Neurotoxicology.

[66] Barinova K., Khomyakova E., Semenyuk P., Schmalhausen E., Muronetz V. (2018). Binding of alpha-synuclein to partially oxidized glyceraldehyde-3-phosphate dehydrogenase induces subsequent inactivation of the enzyme. Arch. Biochem. Biophys..

[67] Albina J.E., Mastrofrancesco B., Reichner J.S. (1999). Acyl phosphatase activity of NO-inhibited glyceraldehyde-3-phosphate dehydrogenase (GAPDH): A potential mechanism for uncoupling glycolysis from ATP generation in NO-producing cells. Biochem J..

[68] Schmalhausen E.V., Medvedeva M.V., Serebryakova M.V., Chagovets V.V., Muronetz V.I. (2021). Products of S-nitrosylation of glyceraldehyde-3-phosphate dehydrogenase: Relation between S-nitrosylation and oxidation. Biochim. Biophys. Acta-Gen. Subj..

[69] Barinova K.V., Serebryakova M.V., Muronetz V.I., Schmalhausen E.V. (2017). S-glutathionylation of glyceraldehyde-3-phosphate dehydrogenase induces formation of C150-C154 intrasubunit disulfide bond in the active site of the enzyme. Biochim. Biophys. Acta-Gen. Subj..

[70] Barinova K.V., Serebryakova M.V., Eldarov M.A., Kulikova A.A., Mitkevich V.A., Muronetz V.I., Schmalhausen E.V. (2020). S-glutathionylation of human glyceraldehyde-3-phosphate dehydrogenase and possible role of Cys152-Cys156 disulfide bridge in the active site of the protein. Biochim. Biophys. Acta-Gen. Subj..

[71] Schuppe-Koistinen I., Moldeus P., Bergman T., Cotgreave I.A. (1994). S-Thiolation of human endothelial cell glyceraldehyde-3-phosphate dehydrogenase after hydrogen peroxide treatment. Eur. J. Biochem..

[72] Rizvi S.H.M., Shao D., Tsukahara Y., Pimentel D.R., Weisbrod R.M., Hamburg N.M., McComb M.E., Matsui R., Bachschmid M.M. (2021). Oxidized GAPDH transfers S-glutathionylation to a nuclear protein Sirtuin-1 leading to apoptosis. Free Radic. Biol. Med..

